# Dynamic Reconstruction of the Nickel Ions’ Behavior in Different Orthodontic Archwires Following Clinical Application in an Intraoral Environment

**DOI:** 10.3390/ma18010092

**Published:** 2024-12-29

**Authors:** Mirela Georgieva, George Petkov, Valeri Petrov, Laura Andreeva, Jorge N. R. Martins, Velizar Georgiev, Angelina Stoyanova-Ivanova

**Affiliations:** 1 Department of Orthodontics, Faculty of Dental Medicine, Medical University of Sofia, St. G. Sofiiski Blvd., 1431 Sofia, Bulgaria; mirela.georgieva@fdm.mu-sofia.bg (M.G.); v.petrov@fdm.mu-sofia.bg (V.P.); laura_andreeva@fdm.mu-sofia.bg (L.A.); 2 Institute GATE, Sofia University, 5 James Bourchier Blvd., 1164 Sofia, Bulgaria; georgi.petkov@gate-ai.eu; 3 Faculdade de Medicina Dentária, Universidade de Lisboa, 1600-277 Lisboa, Portugal; 4 LIBPhys-FCT UID/FIS/04559/2013, 1600-277 Lisboa, Portugal; 5 Unidade de Investigação em Ciências Orais e Biomédicas (UICOB), Grupo de Investigação em Bioquimica e Biologia Oral (GIB-BO), 1600-277 Lisboa, Portugal; 6 Centro de Estudo de Medicina Dentária Baseada na Evidência (CEMDBE)—Cochrane Portugal, Faculdade de Medicina Dentária, Universidade de Lisboa, 1600-277 Lisboa, Portugal; 7 G. Nadjakov Institute of Solid-State Physics, Bulgarian Academy of Sciences, 72 Tzarigradsko Chaussee, 1784 Sofia, Bulgaria; velizar@issp.bas.bg

**Keywords:** orthodontic alloys, nickel ions, dynamics analysis

## Abstract

**Rationale**: Orthodontic archwires undergo chemical and structural changes in the complex intraoral environment. The present work aims to investigate the safe duration for intraoral use (related to the nickel release hypothesis) of different types of nickel-containing wires. By analyzing how the nickel content (NC) varies over time, we aim to provide practical recommendations for the optimal use of said archwires. **Materials and Methods**: Our analysis focuses on the following nickel-containing archwires: stainless steel, Ni-Ti superelastic, heat-activated NiTi and CuNiTi, and multi-force archwires. The studied archwires of each type were divided into three groups: group 1, as received; group 2, retrieved after intraoral exposure for less than 6 weeks; group 3, used for more than 8 weeks. To assess NC, measurements using scanning electron microscopy (SEM), energy-dispersive X-ray (EDX), and laser-induced breakdown spectroscopy (LIBS) were performed in multiple regions of each wire. Statistical analysis of the measured values using one-way ANOVA and multiple group comparisons showed significant differences in nickel content between groups. The dynamic behavior of the statistical results for NC was then modeled using logistic regression and fitted with cubic splines. **Conclusions**: The proposed behavior model, with further refinement, could enable orthodontists to make informed, patient-specific decisions regarding the safe and effective use of orthodontic floss. The overall conclusion of the study is that due to stability, SS-CrNi, HA-Ni-Ti with Cu, and TriTanium^TM^ are suitable for long-term use, and due to higher nickel release, Ni-Ti-Superelastic, HA-Ni-Ti without Cu, and Bio-Active^TM^ are better for short- to medium-term use.

## 1. Introduction

The nickel release (NR) concept has recently received much attention [1,2,3]. Thus far, research investigations have not been able to support or refute the concept that individuals utilizing orthodontic wires containing nickel (Ni) should be considered part of a high-risk category [4,5]. However, since no theory supports the body’s absorption of nickel, it is acceptable to assume that humans should not come in contact with nickel compounds in excess of a particular threshold for health-related reasons. The aim of the present study is to reconstruct the dynamic behavior of nickel ions in different orthodontic archwires following clinical application in an intraoral environment.

In orthodontic treatment with fixed appliances, the most commonly used Ni-containing archwires are made of stainless steel (SS), nickel–titanium (Ni-Ti), or copper–nickel–titanium (Cu-Ni-Ti).

The archwires made from these alloys are subjected to chemical and electrochemical reactions occurring in the mouth, resulting in the breakdown of existing and the formation of new chemical compounds. Orthodontic treatments using NiTi alloys face two main challenges: corrosion and friction, which may lead to nickel release (NR) and weakening of the material. The oral environment accelerates this degradation due to rapid changes in temperature and pH, the presence of cavities and microorganisms, high humidity, and constant mechanical forces [6,7]. All these factors contribute to the faster aging of orthodontic materials, affecting their surface morphology, structure, mechanical properties, and biocompatibility [4,8,9].

Due to the wide variety of orthodontic archwires on the market and aggressive advertising of their unique qualities, the orthodontist faces the dilemma of which archwire to choose and how long it can last in the oral cavity without losing its healing and physicochemical properties. The literature does not sufficiently address these issues that concern clinicians when choosing the most appropriate archwire.

The problem of estimating the relative probability of nickel absorption in the body is not new. Many studies use a variety of methodologies to provide a global or partial answer to the question. Of course, each time, the question is asked in a methodology-specific way. There are two main concepts (ideas) for research: (A) an in vivo study that measures the impact of a particular agent on different types of orthodontic wires for a specific time; (B) an in vitro study that measures the amount of metal ions released from different types of orthodontic wires as a result of their stay for a specific time in an artificial environment resembling natural, for example, artificial saliva. Each study uses different methods for measuring NC. Normally, type A studies do not evaluate the system dynamics. In contrast, the type B) studies end up with dynamic behavior.

A recent example of a type A study is [10]—in vivo study to compare the extent of corrosion on the surface of nickel–titanium (NiTi) wires in various mouthwashes. The patient groups are created according to the type of mouthwash tested. The orthodontic wires were tested for corrosion after 30 days of use. An example of a type B study is [11], which focuses on quantification and comparison between the metal ions released from different bracket-wire combinations and to assess their cytotoxicity in artificial saliva for 1 week, 2 weeks, and one month. More similar methodologies are employed in recent works [12,13,14,15,16,17].

As can be seen, the number of recent works on the subject using in vitro (type B) methodology significantly exceeds the number of studies using in vivo (type A) methodology.

The present work differs methodology from traditional studies in the following way: It analyzes NC after the archwires have been retrieved from the patient. Existing methods (as mentioned above) provide measures while the orthodontic materials are still in use or offer in vitro results or in silico simulation. We aim to provide a general picture of NC dynamics in an in vivo study and prescribe general recommendations for optimal use duration.

Our central hypothesis is that the change in the amount of nickel in the surface layer of each type of orthodontic archwire depends on two main parameters: the type of archwire and the time of intraoral use. The remaining parameters essential for the result (such as the pH, dietary habits, saliva quality of the specific patient or group of patients, lifestyle, and harmful habits) are averaged using the patient’s and wire’s selection method.

The chosen approach does not mean we underestimate the specific combinations of values of the abovementioned parameters, which are essential for the final result. In vitro studies of particular cases (which may cancel out when averaging data obtained by random selection) are necessary and valuable. That is why the orthodontist should also consider them when determining the therapy.

In general, the present work uses a “random” selection of patients—in the sense of the least restrictions on their choice, as the locations of NC measurement on each arch were chosen pseudo-randomly by visually determining the areas of the arch with the most significant corrosion. Nickel content for the different wires is measured using energy-dispersive X-ray spectroscopy (EDS) and scanning electron microscopy (SEM) techniques. For each of the examined wires, the NC has been determined at several points along the wire.

Summary statistics. Our null hypothesis is that the nickel content (NC) remains constant regardless of the duration of use or the material of the wire. We will show that the NC changes statistically significantly for different types of wires and intraoral use duration.

The dynamics of statistically significant results in NC are modeled in two ways: (1) by logistic regression, depending on two parameters, namely, the center (indicating the moment of the maximum rate of change) and the slope (indicating the acceleration of change) and (2) by cubic spline approximation. Unlike logistic regression modeling, consistent with the three stationary regions separated and defined during data collection and averaging, spline approximation gives a general vision of the process.

### 1.1. References to Related Quantitative Works

The SS archwires most commonly used in orthodontic practice are made from a stainless steel alloy and can be cobalt–chromium–nickel and chromium–nickel SS. Chromium–nickel SS contained 71% iron, 18% chromium, 8% nickel, and less than 0.2% carbon. The most commonly used alloy in modern medicine, classified by the American Iron and Steel Institute (AISI), is Type 316L, which contains molybdenum, making it more corrosion-resistant than AISI Type 302 or 304, which are used for manufacturing orthodontic materials [18,19,20]. Ni-Ti superelastic archwires have the following composition: nickel—54–55%, titanium—43–44%, and cobalt—1.6–3% [19,20,21]. Clinical classification according to specific transitional temperatures divided Ni-Ti wires into four groups: I group—work-hardened; II group—superelastic (austenitic active); III group—thermodynamic (martensitic active) with or without the addition of copper; IV group—graded thermodynamic (multi-force) [22,23]. Multi-force orthodontic archwires are made from a Ni-Ti alloy but feature three zones of varying hardness along their length. These regions include the posterior region, where hardness is greatest; the premolar region, where hardness decreases; and the anterior region, where it is the lowest. Consequently, the forces released differ across the regions, with the greatest force in the posterior, reduced in the premolar, and the weakest in the anterior [24,25]. Small changes in the chemical composition of the regions lead to significant changes in the mechanical and thermal properties of the archwires: A 0.9% variation in Ni content (anterior region: 55.2% and posterior region: 56.1%) results in a change in the initial martensitic temperature Ms by 7.8 °C (in the anterior region, Ms = 34.6 °C, and in the posterior region, Ms = 26.8 °C) [26]. The elemental analysis of copper-containing thermodynamic Ni-Ti archwires (Cu-Ni-Ti) shows an approximate composition of 44% nickel, 51% titanium, small amounts of copper (5%), and 0.2–0.3% chromium along their length [27].

### 1.2. Article Outline

The rest of the paper is organized as follows: Section 2 introduces the types of archwires that are examined, as well as NC measuring techniques, statistical tests, and the model for NC dynamics. Section 3 discusses the findings of our work. In Section 4, we compare our results with other studies and provide a list of practical recommendations for working with each type of wire material. Section 5 concludes this work.

## 2. Materials and Methods

### 2.1. Materials and Study Design

To fulfill the study objective, a sample of 293 patients undergoing orthodontic treatment with nickel-containing archwires for up to 6 weeks and over 8 weeks of use was initially reviewed. The study was conducted among the Bulgarian population—Caucasian type. The selection of patients included in the study was determined by formulating the selection criteria.

(a) Patients with formed permanent dentition (these are patients with all grown permanent teeth). There are two types:
  - Over 14 years old—patients with incomplete growth and patients in the “young individual” stage—up to 44 years old (these patients have completed development but with stable bone and no diseases).
(b) Patients are entirely sanitized—without caries and gingivitis, i.e., all possibilities for inflammatory processes in the oral cavity that can affect the mouth’s pH are avoided.
(c) Patients are selected to be non-smokers and not to take aggressive drinks, according to their data.
(d) All patients are clinically healthy.

From this group, 168 patients, each requiring at least six additional months of treatment with fixed appliances, were selected for analysis and instructed on maintaining rigorous oral hygiene. All orthodontic patients have completed an “Informed Consent”, which is mandatory for all health facilities in Bulgaria.

In total, 188 orthodontic archwires were retrieved from these patients and systematically categorized based on type, cross-sectional dimensions, and duration of intraoral placement. The archwires reviewed in the study are presented in Table 1.

The reviewed archwires, for brevity, will be referred to in the text as follows: SS CrNi, “NiTi”, HA without Cu, HA with Cu, TriTanium^TM^, and Bio-Active^TM^ (as received, retrieved up to 6 weeks, retrieved more than 8 weeks).

The clinical procedures were carried out in compliance with the ethical principles outlined in the Declaration of Helsinki by the World Medical Association, as well as the Ministry of Health’s regulations for Good Clinical Practice. Informed consent was obtained from all participants prior to their involvement in the study.

It is important to note that no patient information (age, sex, medical history, race, saliva PH, smoking status, etc.) has been collected for the purposes of this study nor has any patient been directly involved. Analysis of the wires was carried out before their use and after a set period of time after their removal.

### 2.2. Methods

#### 2.2.1. Data Collection

The archwires that we examined were collected during routine orthodontic patient visits. After retrieval, the wires were immersed in a disinfectant solution for 30 min and then cleaned thoroughly using a cotton swab alongside a 95% alcohol solution to remove any unwanted deposits and food debris. Afterward, each wire was sealed in a plastic bag together with its specific datasheet.

#### 2.2.2. Data Measurements

To determine the elemental composition of the studied archwires, we used scanning electron microscopy (SEM)/X-ray dispersive analysis (EDX) and laser-induced breakdown spectroscopy (LIBS).

EDX is a surface analysis technique for determining elemental composition, and LIBS provides a qualitative, on-the-surface, and in-depth determination of the elemental composition (including trace elements). This information is required to model the nickel dynamics. The physicochemical tests were carried out at multiple sites along the length of each reviewed orthodontic archwire. The LIBS is used as a control (double-check) measurement of the SEM/EDX measurements of the relative amount of nickel on the arc surface in weight percentages.

#### 2.2.3. Scanning Electron Microscopy (SEM)/X-Ray Dispersive Analysis (EDX)

The semi-quantitative analysis of the elemental composition and the surface morphology of the archwires was determined using an SEM/EDX apparatus. For the determination of the elemental composition a Bruker Esprit 1.8 (Bruker, Billerica, MA, USA) and a Bruker Esprit 1.82 (Bruker, Billerica, MA, USA) systems with an accelerating voltage of 20 kV and a relative error 0.5–1 wt. % were used. The surface morphology was determined with various SEM systems—Zeiss EVO MA-15 (Zeiss, Oberkochen, Germany) scanning electron microscope with LaB6 cathode on the polished cross-section samples, a FEI Nova NanoSEM 230 (FEI, Hillsboro, OR, USA) microscope, equipped with Schottky field emission gun, and a ZEISS FESEM Ultra 55 (Zeiss, Oberkochen, Germany) scanning electron microscopes with an acceleration voltage of 4 and 20 kV.

#### 2.2.4. Laser-Induced Breakdown Spectroscopy (LIBS)

LIBS was used for qualitative determination of the elemental composition (including trace elements) using a LIBSCAN25+ system (Applied Photonics Ltd., Skipton, UK). The system contains a Q-switched Nd–YAG pulsed laser operated at 1064 nm with a 5 ns pulse duration. The laser beam was focused on the sample by a 25 cm focal length lens. Four spectrometers are contained in the experimental console, which covers a spectral range of 185 to 785 nm with a resolution of 0.01 nm. A special LIBSoft V8.2.0 software (Applied Photonics Ltd., Skipton, UK) was used to analyze the registered data.

#### 2.2.5. Statistical Analysis

For each measurement on the wire, we have compared the quantitative measurements of the nickel content for the three (separated by the duration of use) groups of wires (as received, retrieved up to 6 weeks, retrieved more than 8 weeks) for each of the considered wire types—SS CrNi, NiTi, HA without Cu, HA with Cu, TriTanium^TM^, and Bio-Active^TM^.

The non-parametric Kruskal–Wallis one-way ANOVA test has been used to study the statistical distributions of the values obtained from the quantitative measures. The significance level of the differences between the groups has been determined by the performance of multi-variance comparison based on the ANOVA test statistics, using Mann–Whitney multiple comparative tests, Bonferroni corrected for group comparison. The results are considered statistically significant if they have reached a confidence level higher than 95% (*p* < 0.05). The numerical results of the non-parametric Kruskal–Wallis one-way ANOVA test are given in an ANOVA table in Appendix A: The numerical results of the one-way ANOVA test.

#### 2.2.6. Approximation of Ni Dynamics

The results from the statistical analysis have been modeled by fitting (minimization of the least squares differences) of sigmoid functions (logistic regression) over time. Mathematically, the described dynamical process represents a Bernoulli differential equation, in which the solution is given by a sigmoid function. A sigmoid function Q(t) is defined for a time interval $∆t=t-t_{0}$ as follows:

(1) $$Q\left(t\right)=\frac{1}{1+e^{-k\left(t-t_{0}\right)}}=\frac{e^{k\left(t-t_{0}\right)}}{1+e^{k\left(t-t_{0}\right)}}$$

where e is the natural logarithm base. Equation (1) represents a family of sigmoid curves with center and slope parameters $\left\{t_{0},k\right\}$. The modeling task is then performed by fitting the curves Q(t) to the averaged data in three time points, corresponding to the three considered stages, and the sigmoid parameters are reconstructed. The motivation behind the choice of this model is in accordance with the general assumption that “the rate of change of NC is proportional to the product of Nickel-potent and Nickel-free compounds quantities”. This rule is common to a very general class of substance decay’s cascade chains such as the radioactive decay, as perhaps the most known example.

In addition to the sigmoid function model, we have prepared a fit based on cubic spline polynomials. We create the spline fit using the averaged data in the three time points. The purpose of using spline polynomials is to provide a more general view of the NC dynamics than the sigmoid curves. The sigmoid model contains more details about the dynamics of the nickel content (such as plateaus in the regions where the time intervals meet). Because of this, the simpler behavior of the spline polynomials can be beneficial to better generalize our findings.

## 3. Results

In recent years, we have published some of the data used in this study [28,29]. Taking the results obtained as a basis, we conducted a methodologically new analysis in the context of a different research topic, which expands and refines previous findings.

**Figure 1:**
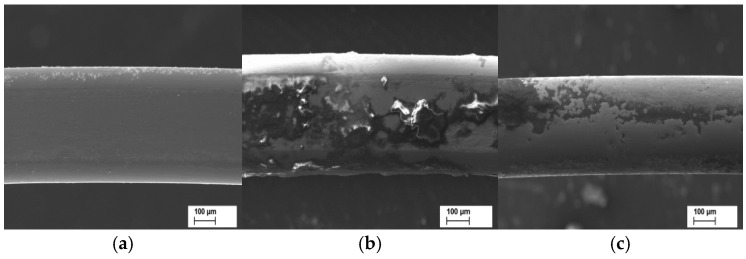
SEM images of an SS archwire. The first wire (**a**) is unused, while (**b**) is taken after 6 weeks of use and (**c**) is an image of a wire that has had intraoral use for more than 8 weeks.

Figure 1 shows an SS archwire under a scanning electron microscope for different durations of intraoral use.

**Figure 2:**
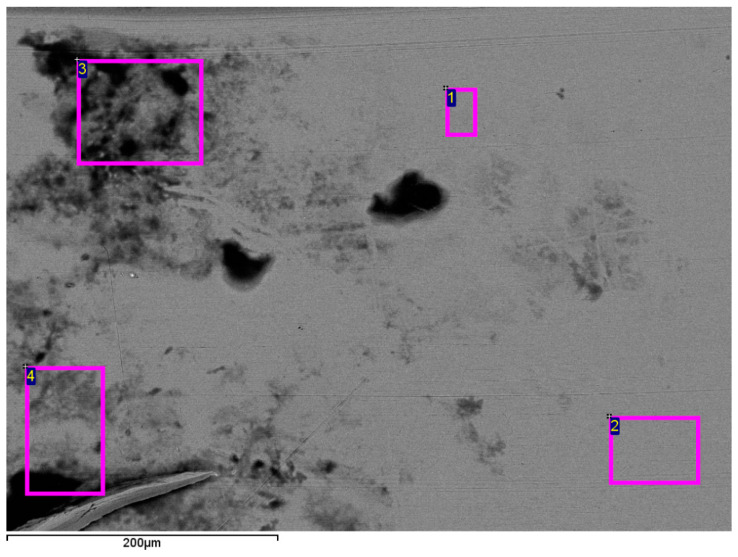
Selection of regions of NC measurements using EDS analysis for a stainless steel archwire that has been used for more than 8 weeks.

Figure 2 shows which areas were for NC measurement based on EDS analysis for the archwire in Figure 1c.

**Figure 3:**
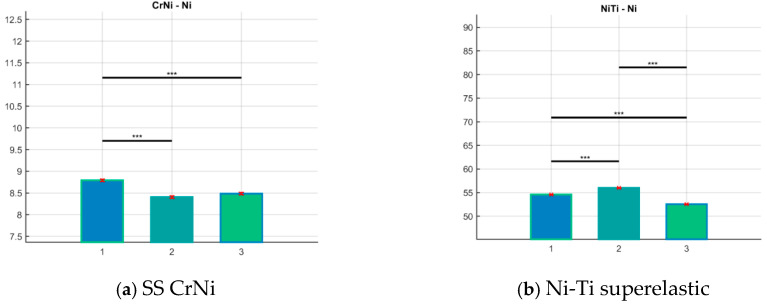

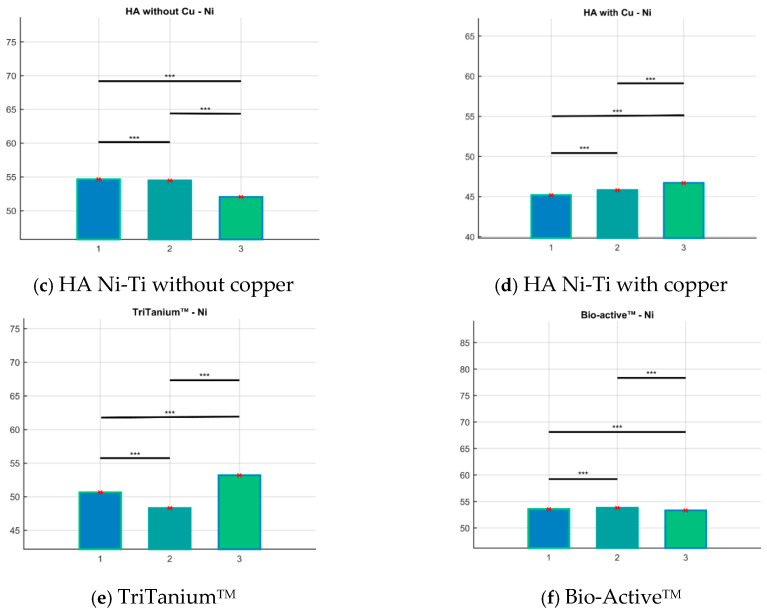
Mean bar plots with error bars for nickel content. The group numbers are placed on the x-axis: as received—#1; retrieved up to 6 weeks—#2; retrieved more than 8 weeks for archwires—#3. Wire composition is presented below each subgraph: (**a**) SS Cr Ni, (**b**) Ni-Ti superelastic, (**c**) HA Ni-Ti without copper, (**d**) HA Ni-Ti with copper, and (**e**,**f**) graded thermodynamic—TriTanium^TM^ and Bio-Active^TM^. The bars represent the mean of the compared nickel-containing archwires for each group (y-axis). The group mean is represented by a bar. Error bars are lines from the top of the bar parallel to the y-axis, representing the uncertainty or SD of the mean value. We marked it with asterisks which in terms of *p*-values are * *p* < 0.05; ** *p* < 0.01, *** *p* < 0.001.

Figure 1 and Figure 2 are illustrations that aim to outline the measurement process. Figure 3 presents the significance level of the differences (Mean bar plots) for nickel content between the main examined types of archwires for the three periods of clinical application.

From Figure 3, one may see that for all of the tested orthodontic archwires, the dynamics of the nickel content are preserved with statistically significant differences in the individual periods, except for the SS CrNi (Figure 3a), where there are no statistically significant differences between the 2nd and 3rd period of clinical application, i.e., the nickel remains statistically constant after the 6th week.

**Figure 4:**
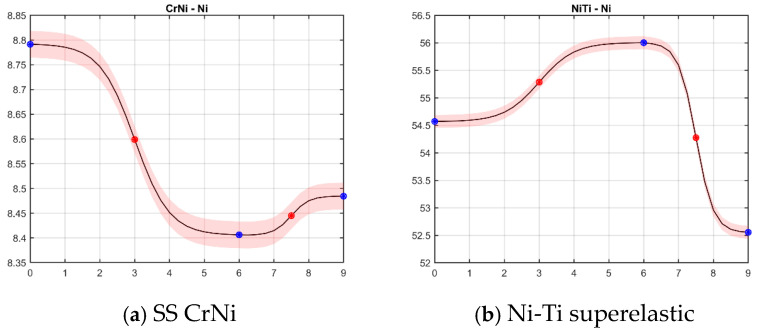

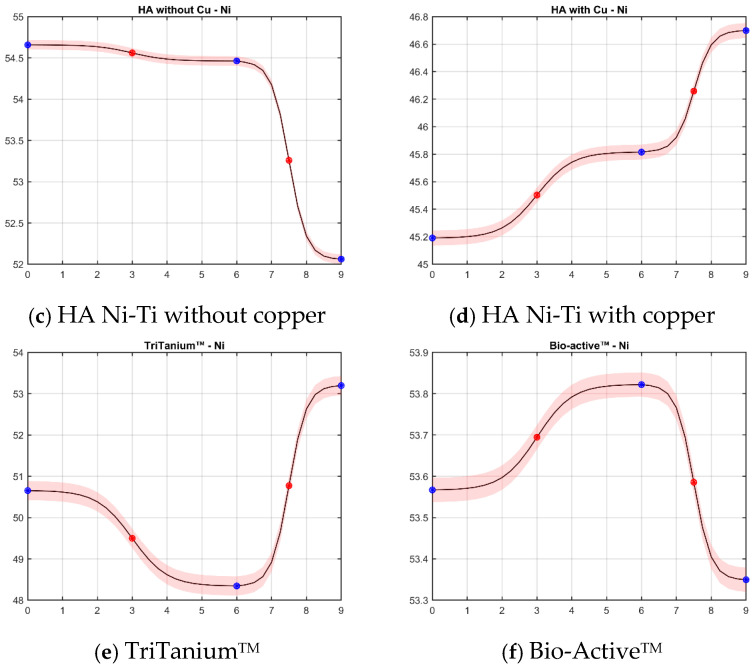
Sigmoid curves approximation: (**a**) SS Cr Ni, (**b**) Ni-Ti superelastic, (**c**) HA Ni-Ti without copper, (**d**) HA Ni-Ti with copper, and (**e**,**f**) graded thermodynamic—TriTanium^TM^ and Bio-Active^TM^. The x-axis shows the duration of intraoral use in weeks. The y-axis shows the Ni mass percentages. The black curve shows the approximated NC dynamics. The blue dots in each panel indicate the data-averaged values for fitting the sigmoid, and the error bars (statistical errors) are patched continuously around the black line. The red dots show the centers of the reconstructed sigmoid. The centers of the sigmoid curves are the points at which the absolute velocity of the dynamic system is most significant. This means that at these points, the relative amount of nickel in the surface layer of the wire increases or decreases most rapidly.

Figure 4 presents a dynamic reconstruction of the nickel ions’ behavior modeled by sigmoid functions (logistic regression) over time.

In Figure 4, the critical points are marked with red dots. According to Pontryagin’s maximum principle, these are the moments at which it should be decided whether to stop or continue the treatment with the archwires. The sigmoid (S-shaped) curves in each graph of Figure 4 represent how nickel content changes as an external variable is applied (intraoral aggressive factors such as bacteria, temperature, pH). These transitions could indicate phase changes or structural transformations in the wire materials, affecting how nickel is retained or released. Most graphs in Figure 4 have three plateau regions (at the beginning, around the 6th week, and at the end), with a steep increase or decrease between them. The plateau phases imply stability in nickel content, while the sharp transition suggests that nickel becomes more reactive or less stable under certain conditions.

**SS CrNi** (Figure 4a) wire has a relatively stable initial nickel content, but as the external factor increases, nickel content drops. This could be due to nickel diffusion or surface oxidation, common in stainless steel when exposed to high mechanical stress.

**Ni-Ti superelastic, HA without Cu, and Bio-Active** (Figure 4b,c,f)—The initial increase could indicate enrichment of nickel at the surface or within the material, potentially due to a reversible phase transformation typical for nickel–titanium alloys. The sharp drop suggests a limit where the material’s structure no longer supports high nickel content and may lead to deterioration of the mechanical properties. The other option is that part of Ni-ions formed new compounds. Indeed, as a compound decays into another substance, which in turn also decays, the relative concentration of the first compound depends on the dynamics of both processes.

**HA with Cu and TriTanium** (Figure 4d,e)—Copper’s presence may stabilize the alloy and initially increase nickel concentration on the surface. However, after a certain threshold, nickel begins to decrease, likely due to phase separation or structural breakdown. TriTanium complex behavior could indicate a multi-phase material structure where nickel migrates within the alloy in response to the external variable.

**Figure 5:**
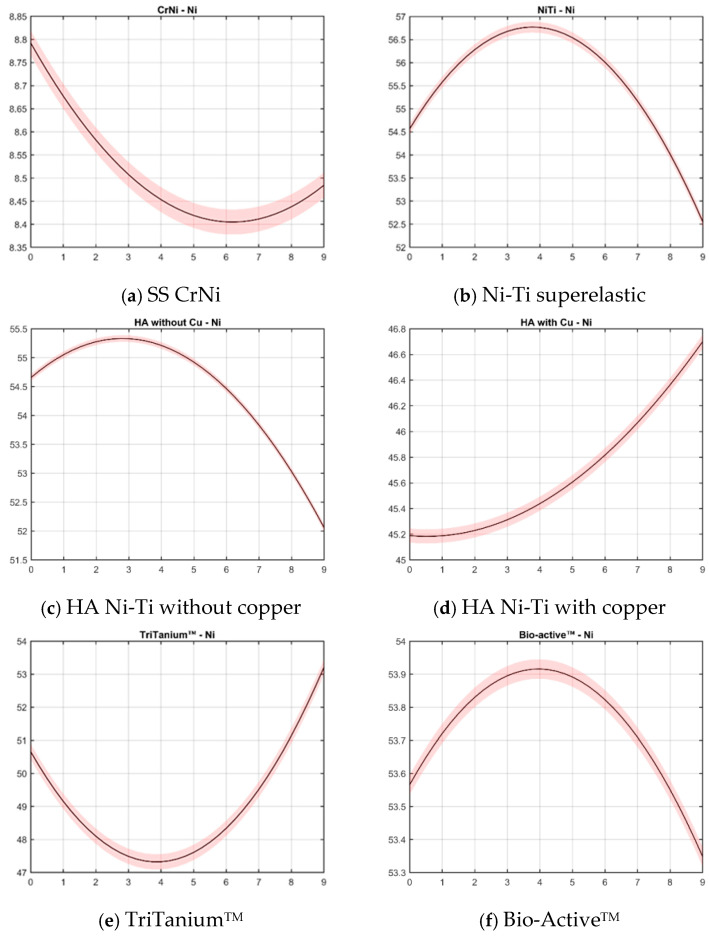
Cubic spline dynamics approximation of Ni content: (**a**) SS Cr Ni, (**b**) Ni-Ti superelastic, (**c**) HA Ni-Ti without copper, (**d**) HA Ni-Ti with copper, and (**e**,**f**) graded thermodynamic—TriTanium^TM^ and Bio-Active^TM^. The x-axis shows the weeks, and the y-axis shows the measured quantities after statistical analysis. The black curve shows the approximated NC dynamics. The error bars (statistical errors) are patched continuously around the black line. The curve shows the general tendency of Ni dynamics, approximated with smooth curves.

Figure 5 illustrates cubic spline dynamics approximations of nickel content for the same orthodontic archwire types. In this context, a cubic spline is a smooth curve made by connecting multiple cubic polynomial segments, which allows for a more flexible representation of changes in nickel content across different points.

According to the curves (Figure 5), three types of behavior could be described:

- Wires with a peak of Ni content around 4 weeks and constant release (Ni-Ti superelastic, HA without copper, and Bio-Active.
- Wires with a drop around 3–4 weeks and constant RELATIVE increasing of Ni-ions (HA with copper, TriTanium).
- Wires with a late drop of nickel ions, around 6 weeks, and then the level of Ni content is kept constant for the long term (SS CrNi).

## 4. Discussion

The importance of the present study lies in its investigation of nickel ion content and release in various orthodontic archwires under intraoral conditions. Orthodontic treatments often require prolonged wire application, and understanding the nickel release profiles of different archwires’ compositions can have significant implications for both patient safety and treatment effectiveness.

### 4.1. Interpretation of the Findings and Comparisons with Previous Studies

Nickel ion release was tested for NiTi, stainless steel, ion-implanted NiTi, and copper-infused NiTi in simulated saliva (pH 5.6–7.0, temperature 36.5 °C) over 7, 14, and 21 days. The highest release occurred on day 7, with daily nickel release values recorded as 0.93 ± 0.04 μg for NiTi, 0.77 ± 0.05 μg for copper-containing NiTi, 0.67 ± 0.02 μg for ion-implanted NiTi, and 0.66 ± 0.02 μg for stainless steel [30]. The problem with this study is that the authors tested the archwires on the 7th, 14th, and 21st day. In average orthodontic treatment, the archwires usually stay more than 30 days. Their findings align with the results of the present study, which shows that SS CrNi releases the smallest amounts of nickel ions, while Ni-Ti superelastic archwires release the largest amounts. We believe that the higher the nickel content in the alloy, the more nickel it releases—also supported by other studies [31]. Applied stress also influences NiTi corrosion; one study found that Copper Ni-Ti^TM^ alloy at 35 °C, in its martensitic phase at mouth temperature, released nickel ions due to localized stress and micro-defect formation under repeated in vivo loading [32]. The examined HA with copper is martensitic active, and the present study shows that the Ni content increases with prolonged clinical application. According to J. Briceño et al. [31], the presence of a martensitic phase in the microstructure improves the corrosion behavior and reduces the ion release by half, which can also be seen in our graphic pictures in Figure 4c.

Food, tobacco smoke, and environmental nickel in the air and water can impact nickel levels in saliva. Studies have reported that the salivary nickel content of patients with orthodontic appliances changes over different stages of treatment, potentially influenced by dietary intake. Another factor affecting the release of nickel ions into saliva is the saliva’s pH level [5,33,34,35,36,37,38]. Kuhta et al. [39] found that lowering salivary pH from 6.75 to 3.5 can amplify metal ion release from orthodontic appliances by as much as 100 times. Additionally, low pH levels decrease the corrosion resistance of dental alloys. Low pH levels also weaken the corrosion resistance of dental alloys. Furthermore, diets high in sodium chloride and acidic sodas introduce corrosive agents into the oral environment [40]. Fluoride-containing products, such as mouthwashes and toothpastes, can also contribute to an acidic oral environment [41,42]. Foods such as vegetables, grains, and cereals are significant sources of nickel [43]. The findings of the current work confirm the findings related to the NC behavior of SS and NiTi archwires, presented in [11].

### 4.2. Practical Recommendations

Based on the data shown in the sigmoid curves, recommendations can be made for the clinical duration of each type of archwire, considering the nickel release patterns over time. Here is an analysis of each archwire type and suggested usage period based on its nickel release characteristics:

SS CrNi (Stainless Steel Chromium–Nickel): The SS Cr-Ni archwire shows an initial drop in nickel content, stabilizing around weeks 7–9. Recommended Duration: Suitable for long-term applications, as it maintains a stable nickel release after the initial period. It can be used for treatments that extend several months.

**Ni-Ti Superelastic:** This wire experiences a peak in nickel release around week 4, followed by a significant drop. Recommended Duration: Best suited for short- to medium-term applications (up to 4–6 weeks), as the peak release could be beneficial during early treatment stages, helping to apply consistent force, but may not be ideal for long-term use.

**HA without Copper: There is a sharp drop in nickel content after week 7, indicating a high initial release that tapers off.** Recommended Duration: Likely suitable for shorter applications (up to 6–8 weeks), given the high initial release. After that, it can be replaced if nickel’s release stability is a concern.

**HA with Copper: This shows a gradual increase in nickel release, stabilizing after week 7.** Recommended Duration: Appropriate for long-term treatments (over several months), as it has a steady release profile that eventually plateaus, reducing risks of excessive nickel exposure.

**TriTanium^TM^: This archwire shows an initial drop in nickel content with a subsequent gradual increase, stabilizing by week 9.** Recommended Duration: Suitable for long-term use, as the nickel content stabilizes over time, making it ideal for applications extending over several months.

**Bio-Active^TM^: This exhibits a peak in nickel release around week 4, followed by a drop and stabilization.** Recommended Duration: May be effective for short- to medium-term treatments (up to 4–6 weeks), with a high release initially to support early treatment forces. It may need replacement if long-term stability is required.

## 5. Conclusions

This study provides the dynamic reconstruction of the nickel ions’ behavior in different orthodontic archwires, including SS CrNi, NiTi, heat-activated with and without copper, and multi-force archwires. The findings highlight that each alloy exhibits unique nickel release patterns influenced by both material composition and environmental conditions. Overall, this study emphasizes the importance of material selection in orthodontics, considering both the mechanical requirements and the biocompatibility concerns associated with nickel ion release. *Limitations*: Our study has certain limitations related to the methodology. Firstly, the analysis was carried out at a group level, which does not account for individual patient-specific differences. As such, the dynamic results in the study should be viewed as tools that can only assist in determining the optimal treatment duration. The exact nickel concentration under which certain safety risks may arise depends on many factors (such as patient age, sex, lifestyle, and eating habits). Our study cannot provide an exact estimate on the individual level.

## Figures and Tables

**Table 1:** Studied nickel-containing orthodontic archwires.

1. Stainless steel archwires	1.1. SS CrNi	
2. Ni-Ti archwires	2.1. Superelastic NiTi (austenitic active)	
	2.2. Thermodynamic, heat activated (HA) (martensitic active)	2.2.1. With copper
		2.2.2. Without copper
3. Graded thermodynamic (multi-force)	3.1. TriTanium^TM^	
	3.2. Bio-Active^TM^	

**Figure A1:**
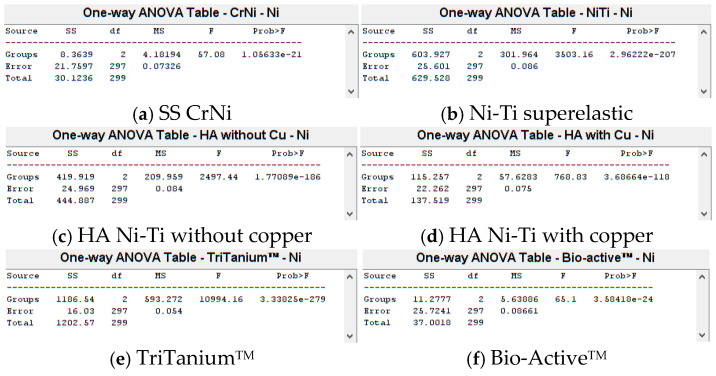
The numerical results of the Kruskal–Wallis one-way ANOVA test. The ANOVA table shows the between-groups variation (Columns) and within-groups variation (Error). SS is the sum of squares, and df is the degrees of freedom. The total degrees of freedom is the total number of observations minus one (the Total row). The between-groups degrees of freedom is the number of groups minus one (the Groups row). The within-groups degrees of freedom is the total degrees of freedom minus the between-groups degrees of freedom (the Error row). MS is the mean squared error, which is SS/df for each source of variation. The F-statistic is the ratio of the mean squared errors. The p-value is the probability that the test statistic can take a value greater than the value of the computed test statistic (Prob > F). The small p-value indicates that differences between means are significant.

## Data Availability

The original contributions presented in this study are included in the article. Further inquiries can be directed to the author.
